# Loss of the Major Phosphatidylserine or Phosphatidylethanolamine Flippases Differentially Affect Phagocytosis

**DOI:** 10.3389/fcell.2020.00648

**Published:** 2020-07-21

**Authors:** Gholamreza Fazeli, Katharina B. Beer, Michaela Geisenhof, Sarah Tröger, Julia König, Thomas Müller-Reichert, Ann M. Wehman

**Affiliations:** ^1^Rudolf Virchow Center, University of Würzburg, Würzburg, Germany; ^2^Imaging Core Facility, Biocenter, University of Würzburg, Würzburg, Germany; ^3^Faculty of Medicine Carl Gustav Carus, Technische Universität Dresden, Dresden, Germany; ^4^Department of Biological Sciences, University of Denver, Denver, CO, United States

**Keywords:** phagocytosis, lipid asymmetry, flippase, phosphatidylserine, phosphatidylethanolamine, extracellular vesicle

## Abstract

The lipids phosphatidylserine (PtdSer) and phosphatidylethanolamine (PtdEth) are normally asymmetrically localized to the cytosolic face of membrane bilayers, but can both be externalized during diverse biological processes, including cell division, cell fusion, and cell death. Externalized lipids in the plasma membrane are recognized by lipid-binding proteins to regulate the clearance of cell corpses and other cell debris. However, it is unclear whether PtdSer and PtdEth contribute in similar or distinct ways to these processes. We discovered that disruption of the lipid flippases that maintain PtdSer or PtdEth asymmetry in the plasma membrane have opposite effects on phagocytosis in *Caenorhabditis elegans* embryos. Constitutive PtdSer externalization caused by disruption of the major PtdSer flippase TAT-1 led to increased phagocytosis of cell debris, sometimes leading to two cells engulfing the same debris. In contrast, PtdEth externalization caused by depletion of the major PtdEth flippase TAT-5 or its activator PAD-1 disrupted phagocytosis. These data suggest that PtdSer and PtdEth externalization have opposite effects on phagocytosis. Furthermore, externalizing PtdEth is associated with increased extracellular vesicle release, and we present evidence that the extent of extracellular vesicle accumulation correlates with the extent of phagocytic defects. Thus, a general loss of lipid asymmetry can have opposing impacts through different lipid subtypes simultaneously exerting disparate effects.

## Introduction

The asymmetric localization of lipids in the plasma membrane is important for many of its barrier and signaling functions (van Meer et al., 2008; Fadeel and Xue, 2009). However, lipid asymmetry is intentionally disrupted during diverse biological processes, including cell division, cell fusion, and cell death (van Meer et al., 2008; Nagata et al., 2020). For example, phosphatidylethanolamine (PtdEth) and phosphatidylserine (PtdSer) are normally enriched on the cytosolic leaflet of the plasma membrane, but dying cells externalize both PtdEth and PtdSer on their surface (Elvas et al., 2017; Nagata et al., 2020). Exposed PtdSer is thought to serve as an “eat-me” signal to induce phagocytosis (Nagata et al., 2020), but several engulfment receptors have been shown to bind both PtdEth and PtdSer (Simhadri et al., 2012; Richard et al., 2015), making it unclear whether exposed PtdEth has a similar or distinct role. Thus, it is important to determine whether a general loss of lipid asymmetry or the exposure of a specific lipid is important for phagocytosis.

Lipid asymmetry is maintained by proteins including aminophospholipid translocases known as flippases that hydrolyze ATP to actively localize specific lipids to the cytofacial leaflet (Kobayashi and Menon, 2018). Initial hints into specific roles for PtdSer and PtdEth during phagocytosis have been provided by studies of two flippases from the *Caenorhabditis elegans* P4-ATPase family: TAT-1 and TAT-5. TAT-1, the ortholog of mammalian ATP8A1 (Zullig et al., 2007), maintains PtdSer asymmetry on the cell surface (Darland-Ransom et al., 2008), but does not play a major role in maintaining PtdEth asymmetry (Wehman et al., 2011). After *tat-1* knockdown, there is an apparent increase in the number of cell corpses (Zullig et al., 2007); indeed, PtdSer exposure on *tat-1* mutant neurons led them to be mistaken for dying cells and cleared (Darland-Ransom et al., 2008). These studies suggest that PtdSer exposure induces cell death or increases phagocytosis.

The flippase TAT-5, orthologous to mammalian ATP9A and ATP9B (Zullig et al., 2007), maintains PtdEth asymmetry in the plasma membrane (Wehman et al., 2011), but does not play a major role in maintaining PtdSer asymmetry (Darland-Ransom et al., 2008; Wehman et al., 2011). TAT-5 and its activator, the large Dopey domain protein PAD-1, prevent PtdEth externalization, and maintain plasma membrane integrity by preventing extracellular vesicle budding (Wehman et al., 2011; Beer et al., 2018). One study found no increase in germline cell corpses after *tat-5* knockdown (Zullig et al., 2007), while another observed that germline cell corpses accumulate after *tat-5* knockdown (Green et al., 2011). Thus, it is unclear whether TAT-1 and TAT-5 have similar roles in preventing cell death or promoting cell corpse clearance.

As previous studies used steady-state assays to test the role of TAT-1 and TAT-5 in cell death and/or phagocytosis, we examined several new models where individual dying cells or cell fragments can be observed from birth to engulfment using time-lapse imaging. For example, the corpse of the second polar body rapidly externalizes PtdSer and is internalized via actin-driven phagocytosis in *C. elegans* embryos (Fazeli et al., 2018). In addition, *C. elegans* embryos phagocytose cell debris called midbody remnants, which are released after cell division and also expose PtdSer on their surface (Chai et al., 2012; Ou et al., 2014; Fazeli et al., 2016). Here, we used these stereotyped models to gain insight into the roles of PtdEth and PtdSer during phagocytosis. We find that disruption of TAT-1 increases phagocytosis of cell debris. In contrast, depletion of TAT-5 or its activator PAD-1 disrupted the phagocytosis of a cell corpse and cell debris, raising the possibility that PtdSer exposure and PtdEth exposure have opposing effects on phagocytosis.

## Results

### Loss of the PtdSer Flippase TAT-1 Leads to Increased Phagocytosis

**FIGURE 1:**
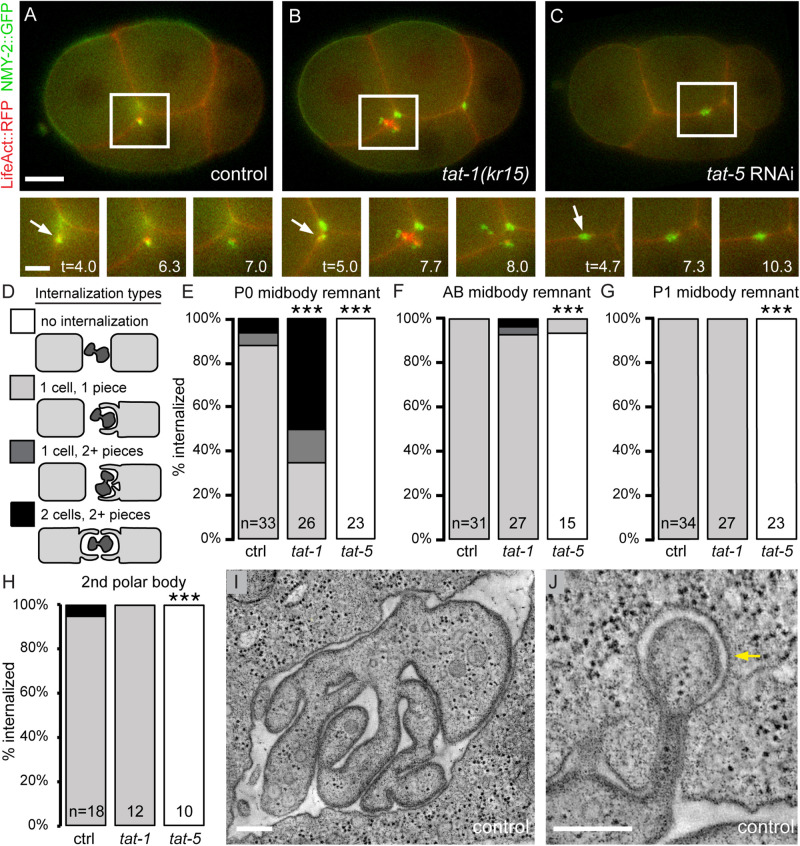
Disruption of the PtdSer flippase TAT-1 increases phagocytosis, while depletion of the PtdEth flippase TAT-5 blocks phagocytosis. **(A)** Before internalization, LifeAct::RFP (red) is enriched on one side of the P0 midbody remnant (arrow) labeled with NMY-2::GFP (green) in a 4-cell control embryo. **(B)** LifeAct::RFP accumulates on both sides in *tat-1(kr15)* mutants during two simultaneous phagocytosis events of the P0 midbody remnant. See also Supplementary Video 1. **(C)** LifeAct::RFP does not accumulate around the P0 midbody remnant in a *tat-5*-deficient embryo and the midbody remnant is not internalized. Lower panels show the P0 midbody remnant before actin accumulation (left), actin polymerization during formation of phagocytic cups (middle), and resolving actin filaments after midbody internalization (right). Similar stages are shown for *tat-5*-deficient embryo, where midbody remnants are not internalized. Scale bar in the main image and insets are 10 μm and 5 μm, respectively. Insets show minutes after 4-cell stage. See also Supplementary Video 4. **(D)** Diagram of modes of internalization observed. **(E–G)** Quantification of midbody remnant internalization in control, *tat-1(kr15)* mutants and *tat-5* RNAi-treated embryos. P0 **(E)**, AB **(F)**, and P1 **(G)** midbody remnants were labeled with NMY-2::GFP or NMY-2::GFP::ZF1. Failed internalization was scored after the 8-cell **(E)** or 15-cell stages **(F–G)**. **(H)** Quantification of internalization of the corpse of the second polar body in control, *tat-1* mutants, and *tat-5* RNAi-treated embryos. Control and *tat-1* polar bodies were labeled with mCh::H2B, *tat-5* RNAi with GFP::H2B. Failed internalization was scored after the 6-cell stage. ***p < 0.001 according to Fisher exact probability test. **(I)** Tomographic slice showing tubulation of the P0 midbody remnant 12.75 min after P0 furrow ingression, short before internalization. Scale bar is 400 nm. See also Supplementary Video 3 showing a three-dimensional model from 3 serial tomograms. **(J)** Tomographic slice of a coated pit (arrow) endocytosing a P0 midbody tubule. Scale bar is 200 nm. Adapted from a tomogram originally published in the Journal of Cell Biology (König et al., 2017).

To investigate the role of PtdSer and PtdEth during phagocytosis, we used fluorescently-tagged non-muscle myosin NMY-2 reporters to label actomyosin in the cytokinetic ring and the resulting midbody remnants released during *C. elegans* embryonic divisions (Figure 1A; Shelton et al., 1999). We discovered that the P0 midbody remnant (derived from the first division of the zygote P0 into the anterior blastomere AB and the posterior blastomere P1) was internalized in two or more pieces in most *tat-1* mutants (65%, Figures 1B,D,E and Supplementary Video 1). Multiple internalization events for the P0 midbody remnant were infrequently observed in control embryos (12%, Figure 1E). To confirm that the NMY-2 reporters were labeling midbody remnants that were internalized in pieces, we also examined a reporter for the centralspindlin protein ZEN-4, which localizes to the spindle midbody and is released in midbody remnants (Green et al., 2013). A ZEN-4 reporter also showed that P0 midbody remnants were internalized in pieces more often in *tat-1* mutants than in controls (84% in *tat-1* vs. 40% in control, Supplementary Figures S1A,B, and Supplementary Video 2). Thus, *tat-1* mutant cells showed an increase in internalization events in addition to increased PtdSer exposure on their surface.

As actin polymerization drives extension of the phagocytic cup around midbody remnants in mammalian cells and *C. elegans* (Crowell et al., 2014; Fazeli et al., 2016), we tested whether the increase in internalization was due to an increase in phagocytosis using a LifeAct reporter to label polymerized actin filaments (Riedl et al., 2008). In control embryos, actin was normally enriched on the ventral side of the P0 midbody remnant before internalization (Figure 1A, *n* = 7), as the ventral endomesodermal precursor cell EMS preferentially internalizes the midbody remnant (Green et al., 2013; Ou et al., 2014; Singh and Pohl, 2014; Fazeli et al., 2016). In cases where an anterior AB descendant internalized the P0 midbody remnant, actin was enriched on the anterior side of the P0 midbody remnant before internalization (Supplementary Figure S2A, *n* = 5). In *tat-1* mutant embryos, actin accumulated on both the ventral and anterior sides of the membrane when two different cells internalized fragments of the P0 midbody remnant (Figure 1B and Supplementary Video 1, *n* = 6). These data reveal the induction of multiple distinct phagocytic events when TAT-1 is disrupted and PtdSer is externalized.

We next wondered whether PtdSer-based signaling was responsible for the stereotyped phagocyte bias for the P0 midbody remnant (Green et al., 2013; Ou et al., 2014; Singh and Pohl, 2014; Fazeli et al., 2016). In control embryos expressing an NMY-2 or ZEN-4 reporter, the P0 midbody remnant is primarily internalized by the posterior cell P1 or its ventral daughter cell EMS (Supplementary Figures S2C,D). Similarly, the majority of P1 or EMS cells internalized fragments of the P0 midbody remnant in *tat-1* mutants (Supplementary Figure S2D), suggesting that the bias for P1 or EMS persists. While only a quarter of anterior AB cells internalize P0 midbody fragments in control embryos, the increase in piecemeal phagocytosis in *tat-1* mutants led to over half of the AB cells acting as phagocytes (Supplementary Figure S2E). Thus, AB cells showed a significant increase in phagocytic activity in *tat-1* mutants.

To test whether the increased phagocytic activity of anterior AB cells was due to increased PtdSer on the surface of *tat-1* mutant cells, we depleted a PtdSer synthase PSSY-1 to reduce global PtdSer levels. This approach was used to suppress intestinal vacuolar defects in *tat-1* mutant worms (Nilsson et al., 2011). Consistently, treating *tat-1* mutants expressing an NMY-2 reporter with *pssy-1* RNAi resulted in significant suppression of phagocytosis of the P0 midbody remnant by AB cells (Supplementary Figure S2E). These observations suggest that the externalization of PtdSer on all cells weakens phagocyte bias, which may be due to biased activation of PtdSer-based phagocytic pathways.

We next tested whether this increase in phagocytosis was unique to the P0 midbody remnant or whether it also occurred to two later midbody remnants labeled with NMY-2 reporters. AB midbody remnants are derived from the second embryonic division of the anterior blastomere AB. In contrast to the P0 midbody remnant, multiple engulfment events were not observed for AB midbody remnants in control embryos (Figure 1F). In *tat-1* mutants, we only infrequently observed internalization of AB midbody remnants in multiple pieces (7%, Figure 1F), despite the strong phagocyte bias for AB midbody remnant uptake by EMS (Ou et al., 2014; Singh and Pohl, 2014; Fazeli et al., 2016). The P1 midbody remnant (derived from the third embryonic division of the posterior blastomere P1) did not show an increase in internalization events in control embryos or in *tat-1* mutants (Figure 1G). Thus, the increase in phagocytic events was observed more frequently for the P0 midbody remnant than for other midbody remnants, suggesting differences in their structure or signaling capabilities.

We next tested whether the observed increase in phagocytosis in *tat-1* mutants would apply to a cell corpse internalized shortly before the P0 midbody remnant (Fazeli et al., 2018). Using a fluorescently-tagged histone (H2B) to label chromosomes, the corpse of the second polar body was internalized in a single engulfment event in both control and *tat-1* mutant embryos (Figure 1H). Similarly, using a fluorescently-tagged PH domain from PLC1γ1 to label PI4,5P2 in the plasma membranes (Fazeli et al., 2018), the second polar body was internalized in a single engulfment event in both control and *tat-1* mutant embryos (Supplementary Figure S1C). Thus, the second polar body was still internalized by only one cell in *tat-1* mutants, suggesting that externalizing PtdSer did not increase engulfment of a dying cell located between two cells of equal phagocytic capacity (Fazeli et al., 2018).

In order to understand how P0 midbody remnants could be engulfed in multiple pieces, we examined their ultrastructure in control embryos. After abscission, the intercellular bridge extends tubules and the P0 midbody remnant becomes a convoluted structure at engulfment stages (Figure 1I and Supplementary Video 3; König et al., 2017). Furthermore, electron densities consistent with endocytic coats are visible engulfing midbody remnant tubules (Figure 1J; König et al., 2017), suggesting that endocytosis of small pieces of the P0 midbody remnant occurs in control cells. Thus, piecemeal uptake of the P0 midbody remnant is common. Taken together, we propose that PtdSer externalization in *tat-1* mutants drives independent phagocytic events to engulf large parts of the P0 midbody remnant.

### Loss of the PtdEth Flippase TAT-5 Disrupts Phagocytosis

As PtdEth is also exposed on the surface of cell corpses (Emoto et al., 1996), we asked whether PtdEth exposure would similarly increase phagocytosis. In contrast to *tat-1* mutants, we observed that midbody remnants labeled with NMY-2 reporters failed to be internalized in *tat-5*-depleted embryos at the expected stages (Figures 1C–G and Supplementary Video 4), similar to phagocytic mutants (Fazeli et al., 2016). The only exception, a single AB midbody remnant, was internalized 25 min later than normal (Figure 1F). This suggests that TAT-5 and/or PtdEth asymmetry are required for the internalization of midbody remnants.

To confirm whether TAT-5 is important to induce formation of a phagocytic cup, we asked whether actin polymerization is induced next to the midbody remnant after *tat-5* knockdown. We had previously observed that LifeAct was not enriched next to the midbody remnant in embryos deficient for the phagocytic signaling protein CED-2/CRK or in mutants for BEC-1/Beclin1, a protein involved in trafficking the engulfment receptor CED-1/Draper/MEGF10 (Fazeli et al., 2016). Consistently, LifeAct enrichment did not occur around the P0 midbody remnant in *tat-5* RNAi-treated embryos (Figure 1C, Supplementary Figure S2B, and Supplementary Video 4, *n* = 8). Thus, TAT-5 and/or PtdEth asymmetry are required for midbody internalization prior to extension of the phagocytic cup.

As loss of PtdSer asymmetry had different effects on the phagocytosis of midbody remnants and cell corpses, we next investigated the effect of PtdEth exposure on cell corpse phagocytosis. The second polar body was labeled with both a PH domain reporter labeling PI4,5P2-containing membranes and an H2B reporter labeling chromosomes to avoid confusion with accumulating extracellular vesicles after *tat-5* knockdown (Wehman et al., 2011). In contrast to control embryos, where the corpse of the second polar body was phagocytosed at the two- or four-cell stage [*n* = 10, (Fazeli et al., 2018)], the second polar body was not phagocytosed by the 24-cell stage in *tat-5* RNAi-treated embryos (Figure 1H, *n* = 10). This suggests that TAT-5 and/or PtdEth asymmetry are required for the phagocytosis of cell corpses as well as midbody remnants.

**FIGURE 2:**
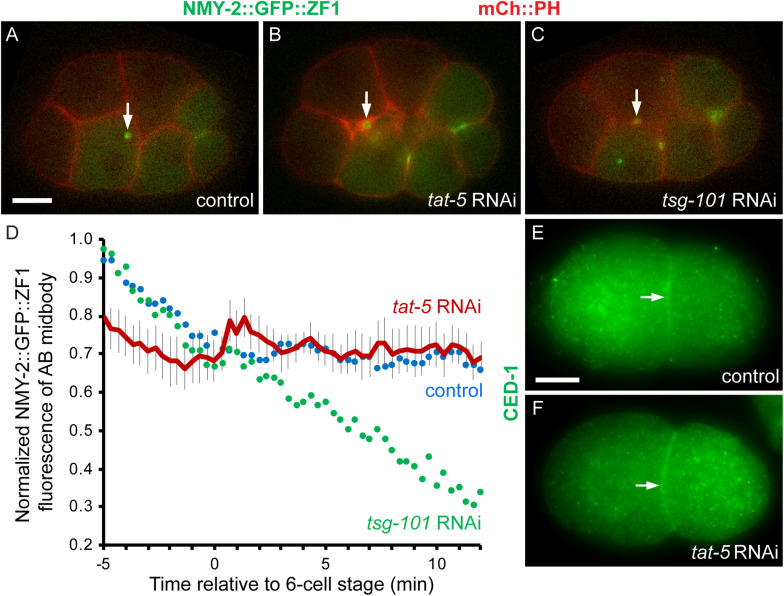
*tat-5*-deficient embryos do not slow abscission or CED-1 trafficking. **(A)** NMY-2::GFP::ZF1 fluorescence (green) labels midbody rings and remnants and is maintained at the AB midbody remnant (arrow) in control 8-cell embryos. mCh::PH labels the plasma membranes (red). **(B)** After *tat-5* RNAi, NMY-2::GFP::ZF1 fluorescence persists on midbody remnants, indicating normal abscission of AB cells and release of the AB midbody remnant. Neighboring extracellular vesicles are labeled with mCh::PH in addition to the plasma membrane. **(C)** NMY-2::GFP::ZF1 fluorescence is reduced in the AB midbody when abscission is delayed by *tsg-101* RNAi. **(D)** Quantification of NMY-2::GFP::ZF1 on AB midbodies and remnants starting at the onset of ZIF-1-mediated degradation, 5 min before the 6-cell stage. Fluorescence is maintained on *tat-*5 RNAi-treated AB midbodies and remnants (*n* = 9). The control and *tsg-101* populations were published previously and are adapted with permission from the Journal of Cell Science (Fazeli et al., 2016). **(E–F)** CED-1 antibody staining localizes to the plasma membrane (arrow) in control and *tat-5* RNAi-treated 2-cell embryos.

Delays in phagocytosis can be caused by delayed or incomplete abscission, for example as a result of disrupting ESCRT proteins (Green et al., 2013; Fazeli et al., 2016). As depleting TAT-5 was previously shown to alter ESCRT localization and occasionally cause multinuclear cells due to cleavage defects (Wehman et al., 2011), we asked whether delayed abscission contributed to the lack of phagocytosis in *tat-5*-depleted embryos. To determine the timing of abscission, we used a degron protection assay to test whether the ZIF-1 ubiquitin ligase could access a ZF1 degron-tagged NMY-2 reporter in the intercellular bridge (Fazeli et al., 2016; Beer et al., 2019). We measured the fluorescence intensity of NMY-2::GFP::ZF1 in the bridge between the anterior daughter cells that form the AB midbody remnant. A steady drop in the fluorescence intensity of the AB midbody starting before the six-cell stage would indicate accessibility and a delay in abscission, similar to depletion of the ESCRT *tsg-101* (Figures 2C,D), whereas stable fluorescence would indicate protection after complete abscission to release the midbody remnant between the resulting daughter cells (Figures 2A,D; Fazeli et al., 2016; Beer et al., 2019). Eight out of nine embryos treated with *tat-5* RNAi maintained NMY-2::GFP::ZF1 fluorescence in the AB midbody remnant (Figure 2B), consistent with abscission defects being rare. Furthermore, the average intensity profile did not drop (Figure 2D, *n* = 9), indicating normal abscission timing. Together, these data reveal that TAT-5 is important for phagocytosis of midbody remnants and cell corpses after abscission but before the formation of the phagocytic cup.

We next asked whether TAT-5 was likely to regulate phagocytosis through a role in receptor trafficking, as TAT-5 orthologs Neo1p and ATP9A are implicated in endocytic recycling (Hua and Graham, 2003; Tanaka et al., 2016). Furthermore, the TAT-5 activator PAD-1 redundantly recycles sorting nexin cargos (Beer et al., 2018), which include the engulfment receptor CED-1 that signals to induce actin polymerization for phagocytosis (Chen et al., 2010). CED-1 localized to the plasma membrane in untreated control embryos (Figure 2E), and still localized to the plasma membrane after treatment with *tat-5* RNAi (Figure 2F, *n* = 14 two- to four-cell embryos), suggesting that the role of TAT-5 in phagocytosis is unlikely to be recycling CED-1 to the plasma membrane. However, we noticed at later stages that CED-1 accumulated in extracellular vesicles between cells (Supplementary Figure S3A, 87%, *n* = 23 six- to 24-cell embryos). The release of CED-1 in extracellular vesicle membranes could lead to reductions in CED-1 levels in the plasma membrane, which could contribute to phagocytic defects. This raised the possibility that TAT-5 inactivation and/or PtdEth externalization could influence phagocytic signaling indirectly by increasing extracellular vesicle release.

### Loss of TAT-5 Activator PAD-1 Also Blocks Phagocytosis

**FIGURE 3:**
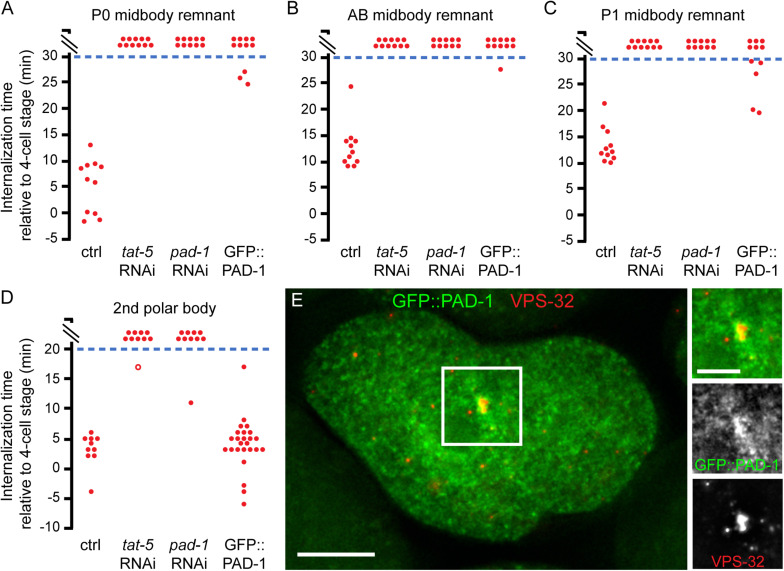
Mutants with exposed PtdEth do not internalize midbody remnants or the second polar body. **(A)** P0 midbody remnants labeled with NMY-2::mCh in untreated control embryos were internalized 5 ± 5 min (mean ± SD) after the 4-cell stage. No P0 midbody remnant labeled with NMY-2::GFP::ZF1 was internalized in embryos treated with *tat-5* or *pad-1* RNAi. Untreated GFP::PAD-1 embryos either did not internalize the P0 midbody remnant labeled with NMY-2::mCh within 30 min past the 4-cell stage or did it significantly later than controls (*p* < 0.001). Each data point represents an embryo. **(B)** AB midbody remnants labeled with NMY-2::mCh in untreated control embryos were internalized 13 ± 4 min (mean ± SD) after the 4-cell stage. No AB midbody remnant labeled with NMY-2::GFP::ZF1 was internalized in embryos treated with *tat-5* or *pad-1* RNAi. Untreated GFP::PAD-1 embryos either did not internalize the AB midbody remnant labeled with NMY-2::mCh within 30 min past the 4-cell stage or did it significantly later than controls (*p* < 0.001). **(C)** P1 midbody remnants labeled with NMY-2::mCh in untreated control embryos were internalized 13 ± 3 min (mean ± SD) after the 4-cell stage. No P1 midbody remnant labeled with NMY-2::GFP::ZF1 was internalized in embryos treated with *tat-5* or *pad-1* RNAi. Untreated GFP::PAD-1 embryos internalized the P1 midbody remnant labeled with NMY-2::mCh significantly later than controls (*p* < 0.001). **(D)** In empty vector controls, the second polar body labeled with mCh::PH::ZF1 is internalized 3 ± 3 min after the 4-cell stage. After *tat-5* or *pad-1* RNAi treatment, embryos failed to internalize second polar bodies within 20 min past the 4-cell stage. mCh::PH and GFP::H2B was used to avoid confusion with extracellular vesicles. The open circle depicts the last frame of a time lapse series that ended before the 20-min cut-off, but where the second polar body was not internalized. GFP::PAD-1 embryos internalized polar bodies labeled with mCh::PH::ZF1 4 ± 4 min after the 4-cell stage, not significantly different from control (*p* > 0.2). **(E)** GFP::PAD-1 (green) colocalizes with ESCRT-III subunit VPS-32 (red) at the P0 midbody remnant in a 3-cell embryo. Scale bar is 10 μm in the main figure and 3 μm in the inset.

As the TAT-5 activator PAD-1 is also required for maintenance of PtdEth asymmetry and inhibition of extracellular vesicle release (Beer et al., 2018), we next asked whether PAD-1 is also required for phagocytosis of cell corpses and cell debris. Control embryos engulfed the P0 midbody remnant within 15 min of P0 abscission (König et al., 2017), while embryos treated with *tat-5* or *pad-1* RNAi failed to engulf P0 midbody remnants labeled with NMY-2 within 30 min of P0 abscission (Figure 3A). Likewise, the AB or P1 midbody remnants were not engulfed in *tat-5* or *pad-1* RNAi-treated embryos at timepoints where all control embryos had engulfed these remnants (Figures 3B,C). Furthermore, polar body corpses labeled with the PH and H2B reporters, which are normally engulfed at the 2- or 4-cell stage (Fazeli et al., 2018), were rarely engulfed by the 7-cell stage after *pad-1* or *tat-5* RNAi treatment (Figure 3D). Together, the *tat-5* and *pad-1* data support that exposure of PtdEth directly or indirectly disrupts phagocytosis.

### Extracellular Vesicle Accumulation Correlates With Phagocytosis Defects

To test whether phagocytic defects were due to increased EV release, we tried to find a strain with a partial loss in PAD-1 function. We had previously knocked GFP into the *N*-terminus of PAD-1 without a linker sequence (Beer et al., 2018). In contrast to strong *pad-1* loss of function, which results in embryonic lethality and sterility (Guipponi et al., 2000; Beer et al., 2018), the GFP knock-in worms were viable and fertile. However, when we examined midbody remnants using an NMY-2 reporter, we found that the engulfment of P0 midbody remnants in GFP::PAD-1 embryos was blocked or significantly delayed (Figure 3A, *n* = 11), similar to *pad-1*-deficient embryos. Phagocytosis of AB midbody remnants was also blocked or significantly delayed in GFP::PAD-1 embryos (Figure 3B), while phagocytosis of P1 midbody remnants sometimes occurred with normal timing, but was mostly significantly delayed (Figure 3C). Despite these phagocytic defects, CED-1 still localized to the plasma membrane in GFP::PAD-1 embryos (Supplementary Figures S3B,C), suggesting that defects in engulfment receptor trafficking were not the cause of the defects in midbody phagocytosis. Interestingly, we noticed that GFP::PAD-1 colocalized with midbody remnants (Figure 3E, *n* = 8), which were labeled with the ESCRT subunit VPS-32 (Morita et al., 2007). These data suggest that the *N*-terminus of PAD-1 could be important for phagocytosis of midbody remnants.

We next asked whether phagocytosis of the second polar body corpse was similarly disrupted by this GFP insertion. We tracked the second polar body with a PH reporter labeling the plasma membrane and found that phagocytosis of the second polar body occurred normally in GFP::PAD-1 embryos (Figure 3D, *n* = 25). Thus, the GFP::PAD-1 knock-in does not disrupt phagocytosis of polar body corpses and appears to be a partial loss-of-function allele, raising the question of whether EV release is increased in this allele.

**FIGURE 4:**
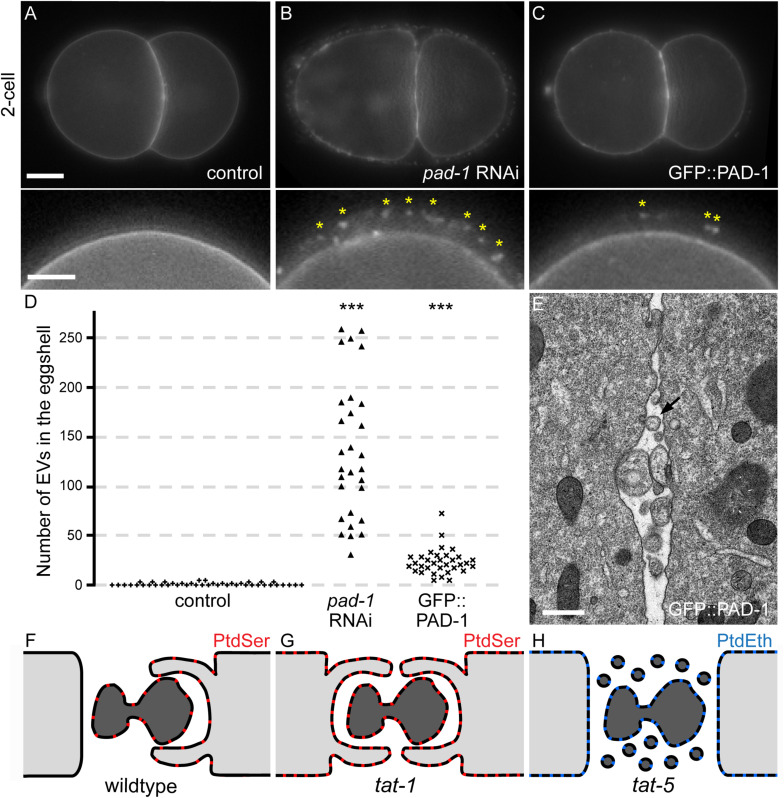
Tagging the *N*-terminus of PAD-1 causes mild extracellular vesicle accumulation. **(A–C)** Images of 2-cell embryos expressing mCh::PH::ZF1 in control **(A)**, *pad-*1 RNAi-treated **(B)**, and GFP::PAD-1 **(C)** embryos. Insets show the magnified eggshell. Asterisks label EVs neighboring the embryo. Scale bar in the main image and insets are 10 μm and 5 μm, respectively. **(D)** The number of EVs in the eggshell *pad-*1 RNAi-treated and GFP::PAD-1 embryos was significantly increased in comparison to control embryo (***p < 0.001). Each data point represents the number of EVs in one embryo. **(E)** TEM of the cell-cell contact from a 2-cell GFP::PAD-1 embryo reveals EVs (arrow) between cells. Scale bar is 500 nm. **(F)** PtdSer is normally externalized on phagocytic cargos as well as phagocytic cells. **(G)** In *tat-1* mutants, PtdSer is externalized on all cells, leading to an increase in phagocytosis. **(H)** In *tat-5* mutants, PtdEth is externalized on all cells, leading to an increase in extracellular vesicle release and a disruption in phagocytosis.

As increased EV release disrupts gastrulation and results in lethality (Wehman et al., 2011), we expected that the GFP::PAD-1 knock-in would not cause a significant increase in EV release from the plasma membrane. We used a plasma membrane-targeted degron reporter to specifically label released EVs (Beer et al., 2018, 2019). In control embryos, labeling with the PH::ZF1 reporter is rarely seen between cells or in the eggshell (Supplementary Figure S3D and Figures 4A,D, *n* = 43), in contrast to *pad-1* knockdown Supplementary Figure S3E and Figures 4B,D, *n* = 29, (Beer et al., 2018)]. In the GFP::PAD-1 strain, the PH::ZF1 reporter did not accumulate between cells (Supplementary Figure S3F, *n* = 33), suggesting that EV release was not increased as strongly as after *pad-1* knockdown. However, an increased number of PH::ZF1 puncta were detected in the eggshell in the GFP::PAD-1 strain (Figures 4C,D), and TEM analysis revealed EVs accumulating between cells (Figure 4E), suggesting that EV release was significantly increased. These quantitative data correlate a >20-fold increase in EVs with disruption of midbody remnant phagocytosis, while a >50-fold increase in EVs correlates with disruption of polar body phagocytosis.

## Discussion

The exposure of aminophospholipids on the exofacial leaflet of the membrane is pivotal for diverse physiological functions, including phagocytosis. Although both PtdSer and PtdEth are exposed on phagocytic cargoes under physiological conditions, such as on cell corpses (van Meer et al., 2008; Nagata et al., 2020), our data reveal that exposure of these phospholipids leads to opposite effects. We discovered that uncontrolled exposure of PtdSer in *tat-1* mutants led cells to increase their phagocytic activity and resulted in a higher number of phagocytic events (Figures 4F,G). In contrast, exposure of PtdEth through the downregulation of TAT-5 or its activator PAD-1 blocked phagocytosis (Figure 4H). As PtdSer exposure can lead to fates other than phagocytosis (Nagata et al., 2020), co-exposure of PtdEth could serve as a “don’t eat me” signal. Thus, the exposure of both aminophospholipids may balance phagocytic signaling to prevent uncontrolled engulfment.

One key open question is whether increased PtdSer exposure on the cargo or engulfing cell is responsible for the increase in phagocytic events observed in *tat-1* mutants (Figure 4G). That actin polymerization usually occurs in the cell that later engulfs the midbody remnant indicates that under normal conditions the engulfment receptors of only one neighboring cell are triggered. During cell corpse engulfment, PtdSer is thought to be directly recognized by the engulfment receptor PSR-1 or a secreted adaptor protein TTR-52 that binds to the engulfment receptor CED-1 (Conradt et al., 2016). Thus, increased exposure of PtdSer on cell corpses and cell debris may boost receptor activation on neighboring cells, leading to more phagocytic events. Indeed, PtdSer externalization appears stronger in *tat-1* mutants than on dying gonads (KBB, unpublished observations). However, PtdSer exposure has also been observed on the surface of engulfing cells (Mapes et al., 2012). Thus, PtdSer exposure on the surface of all neighboring cells in *tat-1* mutants could allow the activation of phagocytic pathways on more cells, resulting in multiple cells engulfing midbody remnants. However, we did not see a complete loss of phagocytic bias in *tat-1* mutants, suggesting that there are other factors in addition to PtdSer-based signaling that determine which cell engulfs cell debris. Furthermore, why PtdSer exposure on the surface of neighboring cells would not increase engulfment attempts on polar body corpses or later midbody remnants is unclear. One possibility is that highly tubulated midbody remnants are more readily severed than spherical corpses, but the relative ultrastructure of different midbody remnants is not known.

In contrast to PtdSer, much less is known about the role of PtdEth externalization. At late stages of cell division, PtdEth is transiently exposed on intercellular bridges and the internalization of PtdEth is thought to be required for abscission to separate cells and release midbody remnants (Emoto et al., 1996), consistent with the observation of occasional cytokinesis defects in *tat-5* mutants (Wehman et al., 2011). It was therefore surprising that abscission was rarely slowed during AB cell division in *tat-5* mutants, especially given the localization of GFP::PAD-1 to midbody remnants. However, our data suggest that TAT-5 and PAD-1 also play important roles after abscission, as they are also required for engulfment of midbody remnants and cell corpses. Thus, it remains to be determined whether PtdEth needs to be internalized to allow phagocytic signaling.

An alternate explanation for the phagocytic defects observed when disrupting TAT-5 and PAD-1 hinges on the accumulation of extracellular vesicles between cells. PtdEth externalization correlates with an increase in extracellular vesicle budding from the plasma membrane (Wehman et al., 2011; Beer et al., 2018), as well as with the phagocytic defects demonstrated here, raising the question how these phenotypes are related. We think it is unlikely that phagocytic defects cause the observed accumulation of EVs, given that the EVs in *tat-5* and *pad-1* mutants are smaller than typical phagocytic cargo, averaging 150–200 nm in diameter. Furthermore, disrupting phagocytic signaling pathways did not result in observable EV accumulation (Fazeli et al., 2016, 2018). Instead, we favor the hypothesis that EV accumulation disrupts phagocytosis. EVs could deplete engulfment receptors like CED-1 from the plasma membrane or physically mask or outcompete phagocytic cargos (Figure 4H). As the mammalian ortholog of TAT-5, ATP9A, also inhibits EV release (Naik et al., 2019), we predict that disrupting TAT-5 and PAD-1 orthologs is likely to disrupt phagocytosis in other species.

P4-ATPases are also involved in membrane trafficking (Andersen et al., 2016) which could explain their role in phagocytosis. We showed that TAT-5 is not required for the localization of at least one engulfment receptor to the plasma membrane, CED-1. However, the partial loss of function of PAD-1 we observed after inserting GFP directly into its *N*-terminus, suggests a possible role for membrane trafficking. The *N*-terminus of the mammalian PAD-1 homolog, Dopey1, binds to the kinesin-1 family of motor proteins and is involved in vesicle transport along microtubules (Mahajan et al., 2019). Thus, as PAD-1 has also been shown to regulate endosomal trafficking (Beer et al., 2018), GFP could spatially interfere with a trafficking function of the *N*-terminal domain of PAD-1. However, why this differentially disrupted the phagocytosis of different cell debris requires further investigation.

In conclusion, our time-lapse studies on four phagocytic cargos revealed new insights into the opposing effects of PtdSer and PtdEth externalization. As four distinct cargos are phagocytosed within a 30-min period during early development, *C. elegans* embryos present a simple genetic model system to tease apart why different cargos rely on distinct signaling pathways. For example, we discovered that PtdSer externalization increased phagocytosis of P0 midbody remnants but did not significantly alter the phagocytosis of other midbody remnants or a cell corpse. Furthermore, this system allows us to ask why different cells have dissimilar phagocytic capacity, as strong biases exist in which cell engulfs each phagocytic cargo (Green et al., 2013; Ou et al., 2014; Singh and Pohl, 2014; Fazeli et al., 2016, 2018). We revealed that PtdSer externalization promoted phagocytosis by anterior cells, but only for a single cargo. These findings emphasize the importance of studying individual phagocytic events to discover mechanisms that would be obscured in bulk or steady-state assays.

## Materials and Methods

### Worm Strains and Maintenance

*Caenorhabditis elegans* strains were maintained on OP50 bacteria according to standard protocol (Brenner, 1974). For a list of strains used in this study and crosses performed to generate strains, see Supplementary Table S1.

### RNAi Experiments

RNAi was performed by feeding dsRNA-expressing bacteria from the L1 larval stage through adulthood with *tat-5* (JA_F36H2.1) or *pad-1* (MV_Y18D10A.15) at 25°C (60–70 h) according to established protocols (Fraser et al., 2000). For the experiments in Figure 3D, worms were fed starting at the L3/L4 stage for 18–24 h. Feeding of *tat-1* mutants with *pssy-1* RNAi (MV_ZC506.3) was performed for 60–93 h from the L1 stage as the treatment resulted in developmental delays and sterility, raising the possibility that the embryos analyzed for Supplementary Figures S2D,E may represent partial knockdown. The *tsg-101* dsRNA was transcribed using T7 RNA Polymerase (Thermo Fisher Scientific) from T7 PCRs of the *tsg-101* RNAi plasmid (MV_C09G12.9), as in Fazeli et al. (2016). 1or 2 mg/ml *tsg-101* dsRNA was injected into the gonad of young adult worms 20–26 h before analysis. Efficiency of *tsg-101* RNAi was judged by a mild delay in internalization timing of the AB midbody remnant. RNAi constructs were obtained from available libraries (Source BioScience).

### Time-Lapse Imaging

Embryos were dissected from gravid adults and mounted in M9 buffer on an agarose pad on a slide. *Z*-stacks were acquired sequentially for green and red fluorescent markers every 20 or 60 s at room temperature using a Leica DM5500 wide-field fluorescence microscope with a HC PL APO 40 × 1.3 NA oil objective lens supplemented with a Leica DFC365 FX CCD camera controlled by LAS AF software. Time-lapse series were analyzed using Imaris (Bitplane). The four- and six-cell stages are defined as the beginning of P1 or both ABx furrow ingression, respectively. Internalization is defined as the first frame where the midbody remnant or second polar body moves away from the plasma membrane, which is likely to closely reflect closure of the phagocytic cup because it correlates with bright actin accumulation. For analysis of second polar body internalization, time lapse series were excluded if another H2B positive object was too close to the second polar body or if the polar body was in two pieces before internalization (*n* = 2 in control and *n* = 3 in *tat-1* mutants).

### Antibody Staining

Gravid worms were dissected in water on a coverslip to release embryos and transferred to 0.1% poly-lysine-coated slides and frozen on dry ice. Eggshells were cracked by flicking off the coverslip and embryos were fixed in methanol before staining with mouse α-CED-1 antibody [1:500, gift of Chonglin Yang (Chen et al., 2010)] or rabbit α-VPS-32 antibody [1:1000, gift of Renaud Legouis (Michelet et al., 2009)], and chicken α-GFP (1:500, 0511FP12 Aves, RRID: AB_2307313). Embryos were then stained with fluorescent secondary antibodies from Jackson ImmunoResearch: Alexa488 donkey α-mouse (Lot: 108424, RRID: AB_2341099), or Cy3 donkey α-rabbit (Lot: 109623, RRID: AB_2307443), and Alexa488 donkey α-chicken (Lot: 108862, RRID: AB_2340375). Slides were counterstained with DAPI to label DNA and mounted using DABCO.

### Image Processing

For clarity, images were rotated and the intensity was adjusted using Adobe Photoshop. Only one *Z*-plane is shown except for Figure 3E, where 5 Zs spanning a region of 1 μm were projected, and Supplementary Figure S2A where 2 Zs were projected (*Z* interval of 1.2 μm). For time lapse videos, data were rotated, projected, colorized, and the intensity adjusted using Imaris.

### Fluorescence Intensity Measurement

Mean fluorescence intensity was measured using ImageJ (NIH) in a circle with area of 0.5 μm^2^ for NMY-2::GFP::ZF1 or 2 μm^2^ for LifeAct::RFP, as described previously (Fazeli et al., 2016). Midbody fluorescence was measured from contractile ring closure until the end of the movie or until the midbody was not distinguishable from the cytoplasm. Fluorescence intensity of the first polar body was measured as an internal control. An exponential decay curve was fit to the polar body data using OriginPro (OriginLab) and used to correct for fluorescence loss due to photobleaching. Embryos were excluded if the P0 and AB midbody remnants were too close to each other (*n* = 1). NMY-2 data are reported as the ratio of the fluorescence intensity of the midbody to the expected value of the polar body after cytoplasmic background subtraction. For LifeAct measurements, normalization was performed using the fluorescence intensity of the cytoplasm.

### EV Counts

Untreated or *pad-1* RNAi-treated WEH260 (mCh::PH::ZF1) or WEH381 (GFP::PAD-1; mCh::PH::ZF1) embryos were imaged on a Leica DM5500. Embryos were analyzed for EVs in Fiji (Schindelin et al., 2012) by counting fluorescent puncta in the eggshell. EVs too close to cells visualized with mCh::PH::ZF1 or DIC were excluded. Thick patches of EVs that occur after *pad-1* RNAi (Beer et al., 2018) were also excluded, likely underestimating the number of EVs.

### Electron Microscopy

WEH381 worms were high pressure frozen and freeze substituted, as described previously (Wehman et al., 2011). 75 nm sections were imaged on a 200 kV JEM-2100 transmission electron microscope (JEOL) equipped with a TemCam F416 4k × 4k camera (Tietz Video and Imaging Processing Systems) running Serial EM software. Tilt series from semi-thick (300–350 nm) serial sections of staged TH155 embryos were collected on a 300 kV Tecnai F30 (Thermo Fisher Scientific, Hillsboro, OR, United States) equipped with a 2 × 2–K charge-coupled device camera (US1000; Gatan) and tomograms were calculated as described (König et al., 2017).

### Segmentation

Serial tomograms (König et al., 2017) were stitched together using eTomo and analyzed and segmented using 3dmod (IMOD; Mastronarde and Held, 2017).

### Statistical Evaluation

Student’s one-tailed *t*-test or Fisher’s one-tailed exact test were used to test statistical significance. In case of multiple comparisons, the Bonferroni correction was used to adjust the statistical significance. Mean ± standard error of the mean is depicted in graphs.

## Data Availability Statement

The raw data supporting the conclusions of this article will be made available by the authors.

## Author Contributions

GF, KB, and AW designed the study, performed, and analyzed experiments. MG quantified fluorescence levels. JK and TM-R contributed electron tomography data that was analyzed by ST. GF and AW wrote the manuscript with input from all authors.

## Conflict of Interest

The authors declare that the research was conducted in the absence of any commercial or financial relationships that could be construed as a potential conflict of interest.
