# Supplementary material for: Loss of the Major Phosphatidylserine or Phosphatidylethanolamine Flippases Differentially Affect Phagocytosis
Source: Front Cell Dev Biol. 2020 Jul 21;8:648. doi: 10.3389/fcell.2020.00648 (PMC7385141; doi:10.3389/fcell.2020.00648)
Supplement: Supplementary file 5 [file Data_Sheet_1.pdf]

Supplementary Material

Loss of the major phosphatidylserine or phosphatidylethanolamine flippases differentially affect phagocytosis

Gholamreza Fazeli[1,2], Katharina B Beer[1], Michaela Geisenhof[1], Sarah Tröger[2], Julia König[3], Thomas Müller-Reichert[3], Ann M Wehman[1,4*]

[1]Rudolf Virchow Center, University of Würzburg, Würzburg, Germany

[2]Imaging Core Facility, Biocenter, University of Würzburg, Würzburg, Germany

[3]Faculty of Medicine Carl Gustav Carus, Technische Universität Dresden, Dresden, Germany

[4]Department of Biological Sciences, University of Denver, Denver, CO, USA

*Email: ann.wehman@du.edu

Supplementary Figures

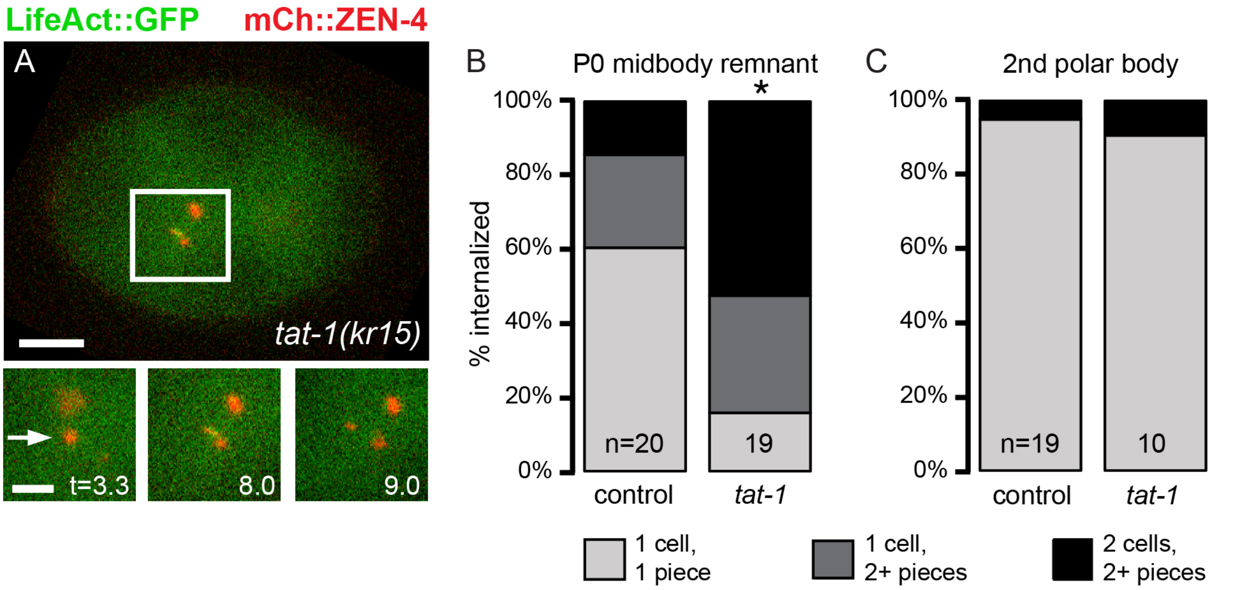

Figure S1. *tat*-1 mutants show increased phagocytosis of P0 midbody remnants, but polar body internalization occurs normally.

A) The P0 midbody remnant (arrow) labelled with a centralspindlin mCh::ZEN-4 reporter (red) is internalized by two cells in *tat-1* mutants. LifeAct::GFP (green) accumulates during internalization. Insets show time after 4-cell stage. Scale bar in the main image and insets are 10 µm and 5 µm respectively. See also Video 2. B) Quantification of internalization of the P0 midbody remnant labeled with mCh::ZEN-4 in control and *tat-1* mutant embryos. *p<0.05 using Fisher exact probability test. C) Quantification of internalization of the corpse of the second polar body labeled with mCh::PH in control and *tat-1* mutant embryos.

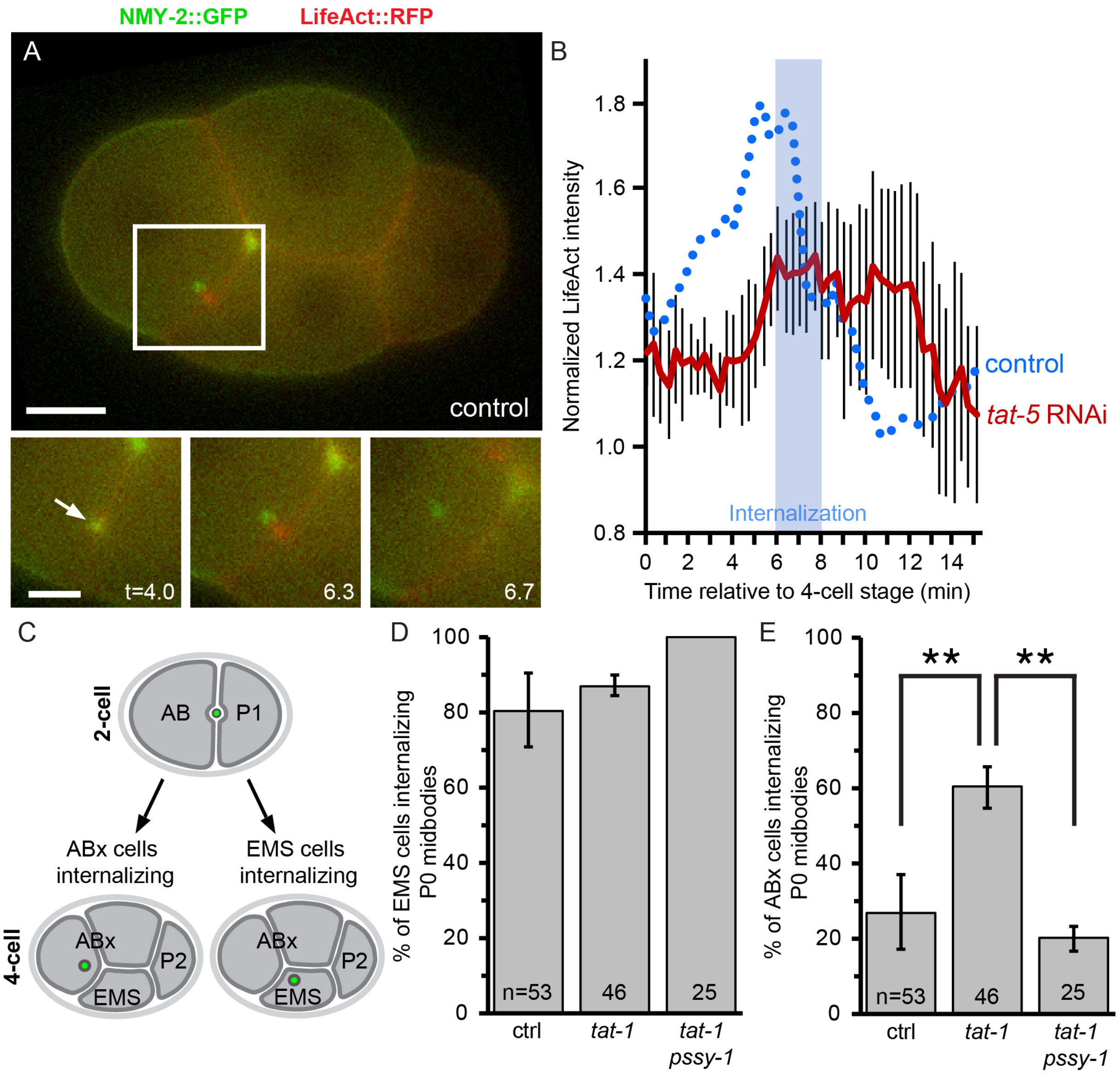

Figure S2. Actin accumulates on the cell that internalizes the midbody remnant, which is influenced by the level of PtdSer externalization.

A) LifeAct::RFP (red, arrow) is enriched on the anterior side during internalization of the P0 midbody remnant (NMY-2::GFP, green) in ABa. Insets show time after 4-cell stage. Scale bar in the main image and insets are 10 µm and 5 µm respectively. B) In control embryos (dotted blue trend line), LifeAct::RFP fluorescence intensity increases gradually around the P0 midbody remnant. After internalization in control embryos (blue box, mean ± SEM, n=13), LifeAct::RFP fluorescence drops. Embryos treated with *tat-5* RNAi (red line) failed to accumulate actin around

the P0 midbody remnant (n=6, p<0.05). The control population was published previously and is adapted with permission from the Journal of Cell Science (Fazeli et al., 2016). C) Model of P0 midbody uptake by anterior ABx cells or the ventral EMS cell. D) P0 midbodies are primarily taken up by EMS in control embryos and *tat-1(kr15)* mutants, even after *pssy-1* RNAi. E) P0 midbody uptake by ABx cells is increased in *tat-1(kr15)* mutants compared to control (**p<0.01, Fisher's exact test). Depleting the PS synthase *pssy-1* reduces phagocytic uptake of P0 midbodies by *tat-1(kr15)* mutant ABx cells (**p<0.01, Fisher's exact test). Control data is averaged from 3 strains: BV113, OD1268, and WEH51. The *tat-1* data is averaged from 3 strains: WEH75, WEH93, and WEH420. The *tat-1 pssy-1* data is averaged from 3 independent L1 RNAi feeding experiments on the WEH93 strain. Mean ± SEM are shown as well as the total number of embryos examined.

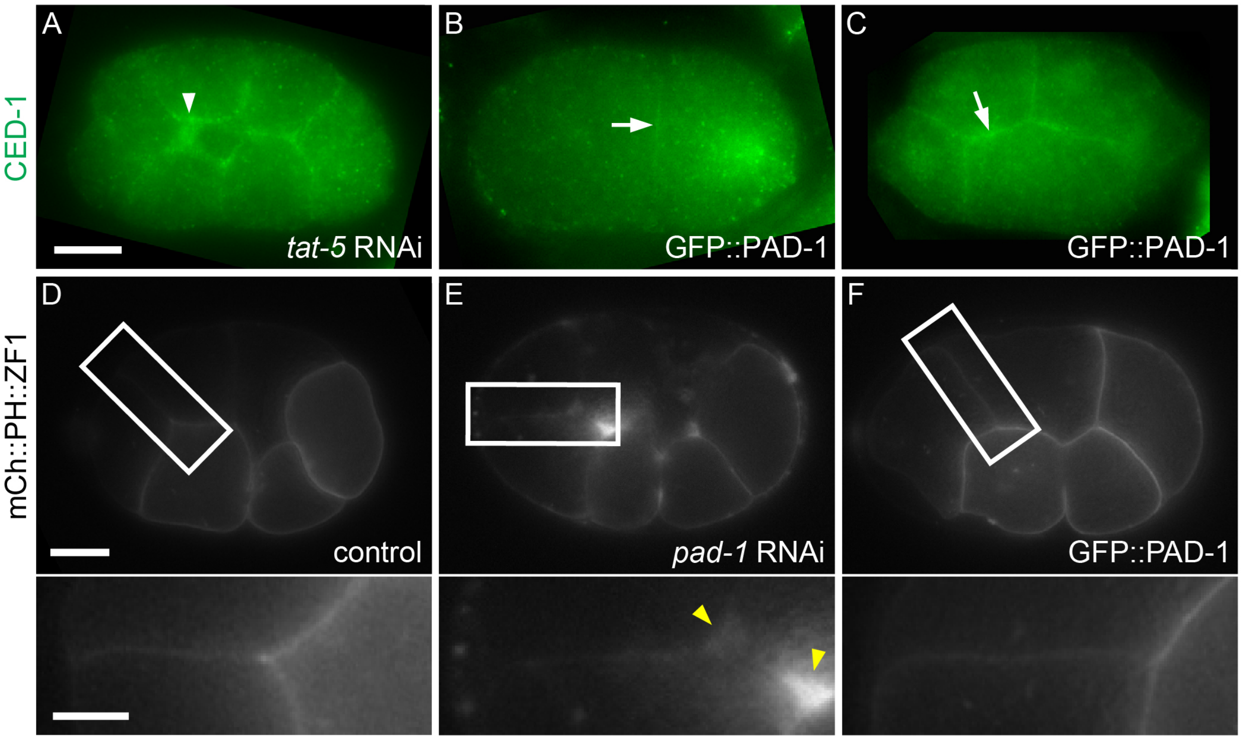

Figure S3. Tagging the N-terminus of PAD-1 does not cause CED-1 mistrafficking or large accumulations of extracellular vesicles between cells.

A) CED-1 staining accumulates in pockets of extracellular vesicles (arrowhead) in a 12-cell *tat-5* RNAi-treated embryo. Scale bar is 10 µm. (B-C) CED-1 antibody staining localizes to the plasma membrane (arrow) in a 2-cell (B) and 6-cell (C) GFP::PAD-1 embryo. D-F) 7-cell embryos expressing mCh::PH::ZF1 in control (D), *pad*-1 RNAi-treated (E) and GFP::PAD-1 (F) embryos. Insets show a magnified cell-cell contact where mCh::PH::ZF1 has been degraded in cells (white box). Arrowheads show pockets containing labeled EVs after *pad-1* knockdown. Scale bar in the main image and insets are 10 µm and 5 µm respectively.

Table S1: Strains used in this study.

**Strain**	**Genotype**	**Source**
N2	Wild type	Brenner, 1974
BV113	*zuIs45[nmy-2p::NMY-2::GFP + unc-119(+)] IV;*<br>*zbIs2[pie-1p::LifeAct::RFP + unc-119(+)]*	Singh and Pohl, 2014
EW0015	*tat-1(kr15:Mos) III*	Ruaud et al., 2009
FT23	*unc-119(ed3) III;*<br>*xnIs8[pJN343: nmy-2p::NMY-2::mCherry + unc-119(+)]*	Nelson et al., 2011
HT1593	*unc-119(ed3) III*	Davis et al., 2009
MCP6	*pad-1(babIs1[GFP]) I*	Beer et al., 2018
OD1268	*ltIs38[pAA1: pie-1p::GFP::PH(PLC1∂1) + unc-119 (+)]*<br>*unc-119(ed3) III;*<br>*hzIs169 [pie-1p::mCherry::ZEN-4 + unc-119 (+)]*	Green et al., 2013
TH155	*ltIs38[pAA1: pie-1p::GFP::PH(PLC1∂1) + unc-119 (+)]*<br>*unc-119(ed3) III; mCherry::β-tubulin*	Konig et al., 2017
WEH02	*ltIs38[pie-1p::GFP::PH(PLC1∂1) + unc-119(+)]*<br>*xnIs8[pJN343: nmy-2p::NMY-2::mCherry + unc-119(+)]*<br>*unc-119(ed3) III*	Fazeli et al., 2016
WEH51	*unc-119(ed3) III; xnIs65 [nmy-2::gfp::zf1 + unc-119(+)] IV;*<br>*ltIs44 [pie-1p::mCherry::PH(PLC1∂1) + unc-119(+)] V*	Fazeli et al., 2016
WEH75	*tat-1(kr15:Mos) III; xnIs65[nmy-2::gfp::zf1 + unc-119] IV;*<br>*ltIs44[pie-1p::mCherry::PH(PLC1delta1) + unc-119] V*	Crossed WEH51 to EW0015
WEH93	*tat-1(kr15:Mos) III;*<br>*zuIs45[nmy-2p::NMY-2::GFP + unc-119(+)] IV;*<br>*zbIs2[pie-1p::LifeAct::RFP + unc-119(+)]*	Crossed BV113 to EW0015
WEH142	*unc-119(ed3) III; zbIs1[pie-1p::LifeAct::GFP + unc-119(+)];*<br>*pie-1p::mCherry::HistoneH2B IV*	Fazeli et al., 2018
WEH153	*tat-1(kr15:Mos) III; zbIs1[pie-1p::LifeAct::GFP + unc-119(+)]*	Crossed WEH142 to EW0015
WEH156	*tat-1(kr15:Mos) III; pie-1p::mCherry::HistoneH2B IV;*<br>*zbIs1[pie-1p::LifeAct::GFP + unc-119(+)]*	Crossed WEH142 to EW0015
WEH231	*unc-119(ed3) ruIs32[pAZ132: pie-1p::GFP::H2B, unc-119(+)] III;*<br>*ltIs44[pie-1p::mCherry::PH(PLC1delta1) + unc-119(+)] V*	Fazeli et al., 2018
WEH260	*wurIs90 [pGF7: pie-1p::mCherry::PH::ZF1 + unc-119(+)] II;*<br>*unc-119(ed3) III*	Beer et al., 2018
WEH381	*pad-1(babIs1[GFP]) I;*<br>*wurIs90[pGF7: pie-1p::mCherry::PH::ZF1 + unc-119(+)] II;*<br>*unc-119(ed3) III*	Crossed MCP6 to WEH260
WEH405	*pad-1(babIs1[GFP]) I; unc-119(ed3) III;*<br>*xnIs8[pJN343: nmy-2p::NMY-2::mCherry + unc-119(+)]*	Crossed FT23 to MCP6
WEH420	*tat-1(kr15:Mos) III; zbIs1[pie-1p::LifeAct::GFP + unc-119(+)];*<br>*hzIs169[pie-1p::mCherry::ZEN-4 + unc-119 (+)]*	Crossed OD1268 to WEH153

Supplementary Video Legends

Video 1. Actin accumulates on both sides of the P0 midbody remnant prior to double internalization in PtdSer flippase *tat-1* mutants.

Time-lapse movie showing the accumulation of actin on both sides of the P0 midbody remnant (arrowhead) prior to its internalization by two neighboring cells in a strain expressing reporters for non-muscle myosin (NMY-2::GFP) to label midbody remnants (yellow) and LifeAct::RFP to label F-actin (cyan). 3 Zs were projected (Z interval 1.2 μm). 5 frames are shown per second.

Video 2. The P0 midbody remnant is internalized by two cells in PtdSer flippase *tat-1* mutants.

Time-lapse movie showing the internalization of the P0 midbody remnant (arrowhead) by two neighboring cells in *tat-1* mutants. The strain expresses a fluorescent reporter for centralspindlin (mCherry::ZEN-4) to label midbody remnants (yellow). 8 Zs are projected (Z interval 1.2 μm) over a single DIC frame. 5 frames are shown per second.

Video 3. 3D model of the P0 midbody remnant before internalization.

3 serial tomograms of the P0 midbody remnant at 12.75 min past furrow ingression were stitched together. Scale bar is 200 nm. P0 midbody remnant membranes were segmented in a rainbow scale changing color every 80 nm for a total of 720 nm in thickness. Neighboring plasma membranes were segmented in beige/brown. 3D model shows multiple tubules stemming from the remnant.

Video 4. Actin accumulation does not occur around midbody remnants that are not internalized in PtdEth flippase *tat-5* mutants. Time-lapse movie showing that actin does not accumulate around the P0 midbody remnant (arrowhead) in *tat-5* mutants. Note that the midbody remnants are also not internalized. The strain expresses fluorescent reporters for non-muscle myosin (NMY-2::GFP) to label midbody remnants (yellow) and LifeAct::RFP to label F-actin (cyan). 3 Zs were projected (Z interval 1.2 μm). 5 frames are shown per second.

Supplementary References

Beer, K.B., Rivas-Castillo, J., Kuhn, K., Fazeli, G., Karmann, B., Nance, J.F., Stigloher, C., and Wehman, A.M. (2018). Extracellular vesicle budding is inhibited by redundant regulators of TAT-5 flippase localization and phospholipid asymmetry. *Proc Natl Acad Sci U S A* 115, E1127-E1136.

Brenner, S. (1974). The genetics of Caenorhabditis elegans. *Genetics* 77, 71-94.

Davis, D.E., Roh, H.C., Deshmukh, K., Bruinsma, J.J., Schneider, D.L., Guthrie, J., Robertson, J.D., and Kornfeld, K. (2009). The cation diffusion facilitator gene cdf-2 mediates zinc metabolism in Caenorhabditis elegans. *Genetics* 182, 1015-1033.

Fazeli, G., Stetter, M., Lisack, J.N., and Wehman, A.M. (2018). C. elegans Blastomeres Clear the Corpse of the Second Polar Body by LC3-Associated Phagocytosis. *Cell Rep* 23, 2070-2082.

Fazeli, G., Trinkwalder, M., Irmisch, L., and Wehman, A.M. (2016). C. elegans midbodies are released, phagocytosed and undergo LC3-dependent degradation independent of macroautophagy. *J Cell Sci* 129, 3721-3731.

Green, R.A., Mayers, J.R., Wang, S., Lewellyn, L., Desai, A., Audhya, A., and Oegema, K. (2013). The midbody ring scaffolds the abscission machinery in the absence of midbody microtubules. *J Cell Biol* 203, 505-520.

Konig, J., Frankel, E.B., Audhya, A., and Muller-Reichert, T. (2017). Membrane remodeling during embryonic abscission in Caenorhabditis elegans. *J Cell Biol.*

Nelson, M.D., Zhou, E., Kiontke, K., Fradin, H., Maldonado, G., Martin, D., Shah, K., and Fitch, D.H. (2011). A bow-tie genetic architecture for morphogenesis suggested by a genome-wide RNAi screen in Caenorhabditis elegans. *PLoS Genet* 7, e1002010.

Ruaud, A.F., Nilsson, L., Richard, F., Larsen, M.K., Bessereau, J.L., and Tuck, S. (2009). The C. elegans P4-ATPase TAT-1 regulates lysosome biogenesis and endocytosis. *Traffic* 10, 88-100.

Singh, D., and Pohl, C. (2014). Coupling of rotational cortical flow, asymmetric midbody positioning, and spindle rotation mediates dorsoventral axis formation in C. elegans. *Dev Cell* 28, 253-267.
